# Follow Up After Hospital Discharge in Older Adult Psychiatric Patients

**DOI:** 10.1192/bjo.2024.519

**Published:** 2024-08-01

**Authors:** Kay Sunderland, Nicholas Rhodes, Andrew Donaldson, Shaina Dillon, Patience Otaniyen

**Affiliations:** 1 NHS GGC, Glasgow, United Kingdom; 2 NHS Lanarkshire, Lanarkshire, United Kingdom; 3 NHS Highland, Inverness, United Kingdom

## Abstract

**Aims:**

To identify if patients discharged from an older adult psychiatric ward were followed up in line with national recommended guidelines. Current National Institute for Clinical Excellence (NICE) guidelines recommend follow up and final discharge letters (FDLs) being available within 7 days of discharge.

**Methods:**

A record search was conducted to identify all patients discharged from one ward during a one year period.

Each patient's notes were reviewed to identify what follow up they had in place and how long it took for this to be implemented. We also examined the time taken for a final discharge letter (FDL) to be made available to their General Practitioner (GP).

**Results:**

We identified 99 patients who were discharged from the ward within the specified period.

The mean time taken for patients to be followed up after discharge was 9.72 days. In 63.16% of cases this follow up was provided by Community Psychiatric Nurses (CPNs), with 51.58% being reviewed in medical clinic. A further 9.47% had their initial follow up with an occupational therapist, 4.21% with a psychologist, 4.21% with the addictions team, 4.21% with care home liaison, 2.11% with social work, 2.11% with continuing care and 1.05% with rehab.

FDLs were sent to GPs, on average, 13.6 days after patients were discharged.

**Conclusion:**

Within our data set a few outlier values markedly increased the mean for both outcomes. Using median figures, average follow up time fell to 6 days, meeting national guidelines, and FDL time fell to 8 days, exceeding recommendations by just 1 day.

Within our department, measures have since been put in place to ensure secretaries are reminding medical staff of the recommended time frames for final discharge letters and it should be noted that an immediate discharge letter (IDL) is routinely sent to GPs containing key clinical information prior to patients being discharged.

The results show that our current practice does fall somewhat short of matching national guidelines and further work should be done to investigate how we can improve standards.

